# Implementation of a Technology-Enabled Diabetes Self-Management Peer Coaching Intervention for Patients With Poorly Controlled Diabetes: Quasi-Experimental Case Study

**DOI:** 10.2196/54370

**Published:** 2024-10-15

**Authors:** Marvyn R Arévalo Avalos, Ashwin Patel, Haci Duru, Sanjiv Shah, Madeline Rivera, Eleanor Sorrentino, Marika Dy, Urmimala Sarkar, Kim H Nguyen, Courtney R Lyles, Adrian Aguilera

**Affiliations:** 1 School of Social Welfare University of California Berkeley Berkeley, CA United States; 2 Pyx Health Tucson, AZ United States; 3 Department of Criminal Justice State University of New York Brockport Brockport, NY United States; 4 MetroPlusHealth New York, NY United States; 5 Division of General Internal Medicine at Zuckerberg San Francisco General Hospital Department of Medicine University of California San Francisco San Francisco, CA United States; 6 Action Research Center for Health Equity, Department of Medicine University of California San Francisco San Francisco, CA United States; 7 Center for Healthcare Research and Policy University of California Davis Health Davis, CA United States; 8 Department of Public Health Sciences School of Medicine University of California Davis Sacramento, CA United States; 9 Department of Psychiatry and Behavioral Sciences University of California San Francisco San Francisco, CA United States

**Keywords:** type 2 diabetes, type 1 diabetes, diabetes experiences, eHealth, mHealth, peer coaching, peer coach, peer support, self-management, social determinants of health, behavioral determinants of health

## Abstract

**Background:**

Patients with diabetes experience worse health outcomes and greater health care expenditure. Improving diabetes outcomes requires involved self-management. Peer coaching programs can help patients engage in self-management while addressing individual and structural barriers. These peer coaching programs can be scaled with digital platforms to efficiently connect patients with peer supporters who can help with diabetes self-management.

**Objective:**

This study aimed to evaluate the implementation of a technology-enabled peer coaching intervention to support diabetes self-management among patients with uncontrolled diabetes.

**Methods:**

MetroPlusHealth, a predominant Medicaid health maintenance organization based in New York City, partnered with Pyx Health to enroll 300 Medicaid patients with uncontrolled diabetes into its 6-month peer coaching intervention. Pyx Health peer coaches conduct at least 2 evidence-based and goal-oriented coaching sessions per month with their assigned patients. These sessions are focused on addressing both behavioral and social determinants of health (SDoH) with the goal of helping patients increase their diabetes self-management literacy, implement self-management behaviors, and reduce barriers to ongoing self-care. Data analyzed in this study included patient demographic data, clinical data (patient’s hemoglobin A_1c_ [HbA_1c_]), and program implementation data including types of behavioral determinants of health and SDoH reported by patients and types of interventions used by peer coaches.

**Results:**

A total of 330 patients enrolled in the peer mentoring program and 2118 patients were considered to be on a waitlist group and used as a comparator. Patients who enrolled in the peer coaching program were older; more likely to be English speakers, female, and African American; and less likely to be White or Asian American or Pacific Islander than those in the waitlist condition, and had similar HbA_1c_ laboratory results at baseline (intervention group 10.59 vs waitlist condition 10.62) Patients in the enrolled group had on average a –1.37 point reduction in the HbA_1c_ score (n=70; pre: 10.99, post 9.62; *P*<.001), whereas patients in the waitlist group had a –0.16 reduction in the HbA_1c_ score (n=207; pre 9.75, post 9.49; *P*<.001). Among a subsample of participants enrolled in the program with at least 2 HbA_1c_ scores, we found that endorsement of emotional health issues (β*=*1.344; *P=*.04) and medication issues (β*=*1.36; *P=*.04) were significantly related to increases in HbA_1c_.

**Conclusions:**

This analysis of a technology-enabled 1-on-1 peer coaching program showed improved HbA_1c_ levels for program participants relative to nonprogram participants. Results suggested participants with emotional stressors and medication management issues had worse outcomes and many preferred to connect through phone calls versus an app. These findings support the effectiveness of digital programs with multimodal approaches that include human support for improving diabetes self-management in a typically marginalized population with significant SDoH barriers.

## Introduction

Diabetes is a significant public health problem that contributes to worsening overall health. Diabetes rates are highest among marginalized (low-income racial and ethnic minority) patients and contribute to high rates of disability and health care costs. Patients with diabetes experience more than double the direct health care expenditures [1], greater comorbidities and disabilities, including depression [2], obesity [3], and increased mortality [4] compared with those without diabetes. Improving diabetes outcomes requires self-management that includes medication management, a healthy diet, and physical activity. Peer support programs can be effective at providing targeted support and increasing motivation to engage in self-management while addressing barriers related to the social determinants of health (SDoH), such as food insecurity and transportation. These programs can be scaled through digital platforms to organize and connect patients with supporters.

Peer coaching interventions can facilitate diabetes self-management and behavior change by addressing both individual and broader SDoH factors that are barriers to self-management. The combination of peer coaching and digital diabetes self-management interventions has the potential to improve health outcomes for patients from marginalized backgrounds. For example, both peer coaching and digitally enabled interventions focused on diabetes self-management are associated with positive behavior change and health outcomes, including reductions in hemoglobin A_1c_ (HbA_1c_) [5-8]. Yet, additional research is needed to elucidate what factors are most salient for patients and how implementation of remote peer coaching diabetes self-management interventions for diverse populations works in real-world settings [9].

Adults who struggle with diabetes may face a variety of challenges with SDoH, such as food insecurity and housing instability [10]. Peer coaches are well-equipped to assist with SDoH barriers related to diabetes self-management as they are often individuals with lived experience representing similar identities as those whom they support. In addition to providing information and skill-building around diabetes self-care strategies (eg, medication adherence, diet, and exercise) [11], they can also provide empathy, concrete strategies, and navigation support for addressing SDoH barriers to diabetes management through referrals and support to cope with or overcome barriers.

Given the existing shortage of licensed health care professionals in chronic and primary care, capacity-building programs focused on peer coaching may help health care providers reach a wider range of their patient population while promoting more individualized and personalized support to these patients [12]. Further, peer coaches may be integrated into health care through task shifting or task sharing, resulting in cost reductions for the system while improving health care delivery and outcomes [6]. Finally, digital health platforms can help scale peer coaching programs more efficiently than in-person programs by reducing geographic and transportation limitations. Private companies developing these platforms may be well-equipped to serve as partners of health care providers and have already been hired to implement digital diabetes interventions, including peer coaching interventions for patients with diabetes.

The purpose of this study is to evaluate the implementation of a technology-enabled peer coaching intervention to support diabetes self-management among patients with uncontrolled diabetes, which was conducted in partnership between a Medicaid health plan and a digital health company. We examine the impact of the program on changes in HbA_1c_, factors associated with changes in HbA_1c_, and implementation outcomes such as program use rates (eg, user engagement measures) in order to develop a comprehensive understanding of program outcomes on patients and health care systems.

## Methods

### Intervention

**Figure 1:**
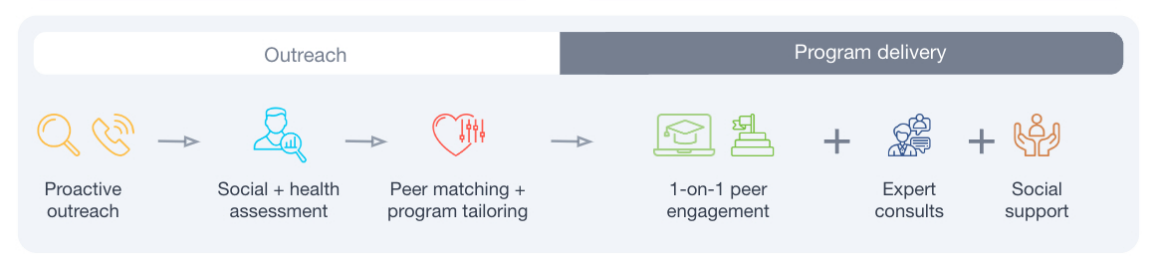
InquisitHealth workflow diagram.

Pyx Health (previously known as InquisitHealth, during the time of the intervention) is a digital health company providing remote peer mentoring services to help patients manage chronic diseases such as diabetes. Pyx Health has developed a services framework characterized by recruiting and training patients who are successful at managing their own health to serve as peer mentors who provide evidence-based and up-to-date educational interventions to patients through telephone or smartphones in both English and Spanish. By partnering with existing health plans and health systems, Pyx Health receives information about patients with uncontrolled diabetes and conducts outreach and enrollment of patients into its programs, matches patients and peer mentors based on shared characteristics (eg, race and language), and conducts a thorough health needs assessment that is used to guide the tailored-content interventions for the patient. Pyx Health also conducts interventions addressing behavioral determinants of health (BDoH) and SDoH that directly impact diabetes self-management. Over the course of 6 months, Pyx Health peer mentors conduct at least 2 evidence-based and goal-oriented coaching sessions per month with their assigned patients. These sessions are focused on addressing both BDoH and SDoH with the goal of helping patients increase their diabetes self-management literacy, implement self-management behaviors, and reduce barriers to ongoing self-care. All peer coaches are trained to follow HIPAA (Health Insurance Portability and Accountability Act)–compliant procedures and use HIPAA-compliant communication methods. A description of Pyx Health workflow and services is presented in Figure 1. Throughout the course of the program, peer mentors are trained to deploy and keep a record of intervention “Tracks” (ie, coach, consult, refer, and share) deployed to best address the needs of their patients.

### Implementation at MetroPlusHealth

MetroPlusHealth, a predominant Medicaid HMO based in New York City, partnered with Pyx Health to enroll 300 Medicaid patients with uncontrolled diabetes into its peer mentoring intervention. A joint approach between these 2 partners took place in 2019 to reach the targeted patient population. MetroPlusHealth case management and quality team members were onboarded to Pyx Health’s Care Coordination platform to receive individual patient escalations. Biweekly meetings between stakeholders were established to oversee the implementation of the Pyx Health program. After the implementation of the program, Pyx Health partnered with the University of California, San Francisco S.O.L.V.E. Health Tech to conduct the evaluation of this program.

### Participants and Recruitment

First, in June 2019 MetroPlusHealth mailed letters and sent an SMS text message to eligible patients (eg, uncontrolled diabetes, HbA_1c_>9%) notifying them about the Pyx Health program. Next, Pyx Health’s outreach team called individual members, and for those reached, eligibility was confirmed (ie, uncontrolled diabetes, English or Spanish speaker, MetroPlusHealth member, and able to participate at least through a landline phone), and if interested, they were enrolled into the program. Pyx Health paused outreach by late August 2019 after enrolling 304 patients into the program. At this time, several patients were already in the recruitment process (eg, scheduled calls), and by early September, 330 patients had enrolled in the peer mentoring program. As the targeted enrollment was met, patients who did not receive an outreach call were part of the “waitlist” group.

On the enrollment call, Pyx Health performed a needs assessment with each patient, which included (1) asking about their behavioral determinants and SDoH that posed barriers to diabetes self-management and ongoing care; (2) matching patients with a peer mentor based on shared lived experience with diabetes, language, race and ethnicity, availability, age, gender, as well as factors like the peer’s capacity, and expertise; (3) scheduling the first call between the patient and their matched peer mentor; and (4) creating a custom program for the patient based on the specific needs, barriers, and challenges identified through the initial assessment using Pyx Health’s platform (which enables both digital and phone engagement).

### Data Sources and Analysis

MetroPlusHealth provided patient demographic data, care use, and clinical data to Pyx Health for the purpose of patient recruitment and data analysis. This included study outcomes data, specifically the patient’s HbA_1c_. Pyx Health collected program implementation data including types of BDoH and SDoH reported by patients. BDoH included personal barriers related to appointments, disease knowledge, disease self-management, emotional health, glucose monitoring, physical activity, healthy eating, medication adherence, co-occurring health conditions, and alcohol use. SDoH included structural barriers to accessing appointments, health literacy, food insecurity, housing instability, glucose monitor, glucose test strips access, medication cost, insurance coverage, and alcohol abuse. We also calculated what types of intervention tracks were used (ie, coach, consult, refer, and share) by peer coaches, as well as user-engagement metrics (ie, phone calls, phone minutes, check-ins, messages exchanged, mailings, and app use frequency).

Pyx Health conducted data analysis while consulting with the University of California, San Francisco S.O.L.V.E. Tech.

We began by conducting *t* test analyses comparing the demographics and HbA_1c_ data between patients enrolled in the program versus those in the waitlist group (ie, not yet contacted for recruitment). The data and outcomes from the patients in the waitlist group serve as a basis of comparison in this study to contextualize the results of the Pyx Health peer mentoring program implementation; however, they do not represent a random control condition.

To analyze changes in HbA_1c_ scores between the waitlist and enrolled groups, individuals needed to have at least 2 HbA_1c_ readings during the study. For the enrolled group, at least 1 HbA_1c_ needed to be 90 days before the specific individual’s program start date. For the waitlist group, since no individual start date was available, at least 1 HbA_1c_ needed to be 90 days before the overall program start date. For both groups, we defined preintervention as 90 days before the program start date, June 19, 2019, and postintervention as 90 days after the program start date through March 30, 2020, when in-person labs were no longer happening consistently due to the COVID-19 pandemic. Finally, we report descriptive statistics for the program implementation data and report results of an exploratory analysis examining factors (eg, demographics, BDoH, and SDoH) associated with changes in HbA_1c_ among the participants in the peer mentoring program.

### Ethical Considerations

This work was approved by the University of California San Francisco institutional review board (#19-28839) as exempt research.

## Results

### Primary Analysis

**Figure 2:**
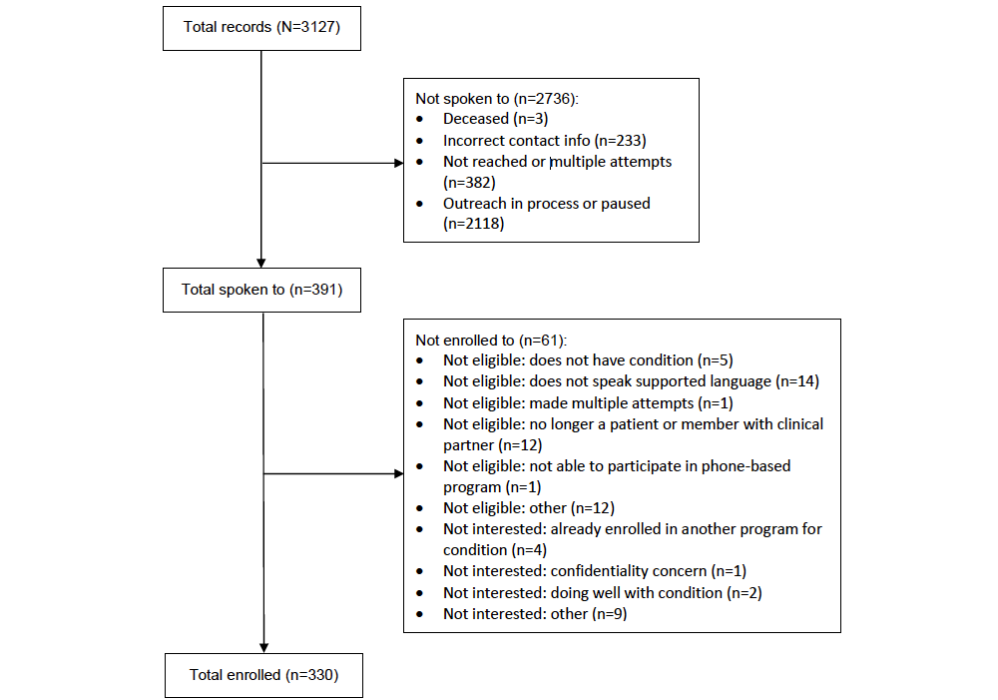
Consort diagram.

Pyx Health received a total of 3127 patient records from its MetroPlusHealth partners. A total of 391 patients were successfully contacted during the phone-based outreach process to meet the enrollment target. At that point, 84.4% (330/391) enrolled in the peer mentoring program, 11.5% (45/391) were not eligible for enrollment, and 4.1% (16/391) were not interested in participating. Of the initial registry list, a total of 618 patients were unreachable and excluded from subsequent analysis. The remaining 67.7% (2118/3127) of patients were considered to be on the waitlist group and used as a comparator. Figure 2 shows a CONSORT (Consolidated Standards for Reporting Trials) diagram with the participant breakdown. For subsequent analysis and when possible, we compared differences between the enrolled and waitlist groups.

First, we examined demographic variables baseline differences across these 2 groups (Table 1), and the results indicated patients who enrolled in the peer mentoring program were older; more likely to be English speakers, female, and African American; and less likely to be White or Asian American or Pacific Islander than those in the waitlist condition. There were no statistically significant differences between the enrolled patients’ HbA_1c_ (mean 10.59, SD 1.79) and those in the waitlist condition (mean 10.62, SD 1.75) at baseline.

Patients enrolled in the peer mentoring program endorsed a variety of behavioral and SDoH challenges to diabetes self-management with the top 3 issues being navigating medical appointments, knowledge about diabetes and disease self-management, and concerns with emotional health (Table 2).

To address these concerns, peer mentors primarily used the intervention tracks of coaching and sharing of resources (Table 3), which accounted for 536 (31.2%) and 889(51.8%), respectively, of all 1717 interventions used.

Finally, patient engagement metrics indicate that on average patients participated in an average of 16.4 calls and 146.3 minutes of intervention by peer coaches during their program participation (Table 4).

**Table 1:** Baseline participant demographics and clinical data.

Participant characteristics		Enrolled (n=330)	Waitlist (n=2118)	*P* value
Age (years), average (SD)		53.35 (9.47)	51.84 (10.95)	.009
Gender (male), n (%)		149 (45.15)	1090 (51.46)	.04
**Race or ethnicity, n (%)**				
	White	10 (3.03)	114 (5.38)	.09
	Black or African American	151 (45.76)	717 (33.85)	<.001
	Asian, Native Hawaiian, or other Pacific Islander	49 (14.85)	422 (19.92)	.04
	Other	64 (19.39)	474 (22.38)	.25
	Hispanic or Latino	27 (8.18)	151 (7.51)	.75
	Unknown	28 (8.48)	207 (9.77)	.52
**Language, n (%)**				
	English	302 (91.52)	1847 (87.2)	.03
	Spanish	25 (7.58)	194 (9.16)	.40
Lives in a disadvantaged zip code, n (%)		82/328 (25)^a^	452/2115 (21.37)^b^	.16
**Hemoglobin A_1c_ data**				
	Baseline hemoglobin A_1c_, average (SD)	10.59 (1.79)^c^	10.62 (1.75)^d^	.81
	Has follow-up hemoglobin A_1c_, n (%)	70 (21.21)	207 (9.77)	<.001

^a^Includes 328 participants.

^b^Includes 2115 participants.

^c^Includes 329 participants.

^d^Includes 1867 participants.

**Table 2:** Patients endorsing behavioral and social determinants of health issues (n=330).

Issue category	Behavioral determinant of health patient (n=330), n (%)	Social determinant of health patient (n=330), n (%)
Appointments	252 (76.4)	240 (72.7)
Knowledge	169 (51.2)	56 (17.0)
Emotional health	174 (52.7)	N/A^a^
Food insecurity	N/A	162 (49.1)
Housing	N/A	149 (45.2)
Glucose monitoring	63 (19.1)	81 (24.6)
Physical activity	144 (43.6)	N/A
Healthy eating	143 (43.3)	N/A
Medication	52 (15.8)	50 (15.2)
Insurance	N/A	68 (20.6)
Alcohol	10 (3.9)	5 (1.5)

^a^N/A: not applicable.

**Table 3:** Intervention tracks used by peer mentors and frequency of use.

Category	Definition	Total (n=1717), n (%)
Coach	Provide evidence-based strategies for addressing the presenting concern through empathy, understanding, and goal-setting	536 (31.2)
Consult	Escalate the issue to the Pyx Health team to triage and escalate concerns to health plan resources (member services, case management, etc) or community-based partners who will proactively engage with the patient for targeted SDoH^a^ concerns	111 (6.5)
Refer	Provide resources, including contact details (website, phone number, addresses, etc), to help patients proactively reach out and engage	181 (19.5)
Share	Provide printed educational materials (mailed to participant), digital content (shared through SMS or smartphone app), and guidance for peer mentor to discuss real-time while on the phone with the participant	889 (51.8)

^a^SDoH: social determinants of health.

**Table 4:** Pyx Health patient engagement metrics (n=330).

Engagement variables	Values, mean (SD)	Range
Phone calls (n)	16.4 (9.4)	2-64
Phone (min)	146.3 (137)	8-826
Check-ins and goals (n)	63.4 (35.4)	5-291
Messages exchanged (n)	12.6 (18.5)	0-127
Mailings (n)	0.2 (0.4)	0-1
App use (0 or 1)	0.2 (0.4)	0-1

### Post Hoc Exploratory Analysis and Results

To compare changes in HbA_1c_ between enrolled participants and the waitlist comparison group, we conducted a post hoc analysis and examined differences between the baseline and postintervention HbA_1c_ by group for patients with at least 2 HbA_1c_ laboratory results (n=277). Patients in the enrolled group had on average a –1.37 reduction in the HbA_1c_ score (n=70; pre 10.99, post 9.62; *P*<.001), whereas patients in the waitlist group had a –0.16 reduction in the HbA1c score (n=207; pre 9.75, post 9.49; *P*<.001).

Finally, to understand factors related to improvement in HbA_1c_ levels, we examined the relationship between demographic variables and endorsement of social and BDoH issues and changes in HbA_1c_ among patients in the enrolled group who had at least 2 HbA_1c_ laboratory results (n=70; Table 5).

We conducted a linear regression analysis with age, gender, and dichotomous (0=no and 1=yes) variables indicating endorsement of the particular issue as predictors of change in HbA_1c_ (Change = post-HbA_1c_ – pre-HbA_1c_). The results of the regression indicate the predictors accounted for 26.5% of the variance in HbA_1c_ changes with the endorsement of emotional health issues (β*=*1.344; *P=*.04) and medication issues (β*=*1.36; *P=*.04) as significantly related to increases in HbA_1c_ (Table 6).

**Table 5:** Demographic variables and endorsement of issues by a subsample of patients with at least 2 hemoglobin A_1c_ (HbA_1c_) scores (n=70).

Variable	Patients with at least 2 HbA_1c_ scores
Age (years), mean (SD)	53.81 (8.65)
Gender (male), n (%)	29 (41.4)
SDoH^a^ (yes), n (%)	59 (84.3)
Appointments BDoH^b^, n (%)	57 (81.4)
Knowledge BDoH, n (%)	43 (61.4)
Emotional health BDoH, n (%)	41 (58.6)
Healthy eating BDoH, n (%)	38 (54.3)
Physical activity BDoH, n (%)	30 (42.9)
Medication BDoH, n (%)	15 (21.4)
Glucose monitoring BDoH, n (%)	14 (20.0)
Alcohol BDoH, n (%)	4 (5.7)

^a^SDoH: social determinant of health.

^b^BDoH: behavioral determinant of health.

**Table 6:** Regression of demographics, endorsed issues, and changes in hemoglobin A_1c_ (HbA_1c_) among patients with at least 2 HbA_1c_ scores (n=70).

Variable	β	SE	*t* test (2-tailed), (*df*)	*P* value
(Intercept)	0.102	1.777	0.058 (58)	.95
Age	–0.035	0.032	–1.095 (58)	.28
Gender (1=male, 0=female)	–0.159	0.541	–0.295 (58)	.77
SDoH^a^	1.432	1.691	0.847 (58)	.40
Alcohol BDoH^b^	1.59	1.072	1.483 (58)	.14
Appointments BDoH	–2.20	1.528	–1.44 (58)	.16
Emotional health BDoH	1.344	0.625	2.151 (58)	.04
Glucose monitoring BDoH	0.648	0.667	0.971 (58)	.33
Healthy eating BDoH	0.052	0.593	0.088 (58)	.93
Knowledge BDoH	–0.381	0.626	–0.609 (58)	.54
Medication BDoH	1.36	0.636	2.139 (58)	.04
Physical activity BDoH	–0.103	0.571	–0.181 (58)	.86

^a^SDoH: social determinant of health.

^b^BDoH: behavioral determinant of health.

## Discussion

This study assessing the implementation of a technology-enabled peer coaching program for patients with uncontrolled diabetes enrolled in a New York City-based managed care plan found that the majority of these patients were from historically marginalized populations (eg, low-income and racial or ethnic minorities) and experiencing high disease burden as measured by average HbA_1c_ >9%. This high level of participation from marginalized and underserved populations is rare in private digital health interventions [13]. Participants in the peer coaching program were more likely to conduct follow-up HbA_1c_ testing compared with their waitlist counterparts (70/330, 21.21% vs 207/2118, 9.77%), which may be attributed to engagement with the peer coaching program and is associated with improvements in glycemic control [14]. In terms of glycemic control, participants had a greater and more significant reduction in HbA_1c_ compared with a waitlist comparison group (n=70; pre 10.99, post 9.62, 12.5% reduction vs n=207; pre 9.75, post 9.49, 1.6% reduction), suggesting the effectiveness of the intervention. In addition, significant predictors of higher HbA_1c_ levels included endorsement of emotional health concerns and medication management issues indicating these issues as prime targets for diabetes self-management interventions. The results of this study add to the literature on the effectiveness of peer coaching [11] and technology-enabled interventions [15] for diabetes self-management among underserved and historically marginalized patients with uncontrolled diabetes.

Historically marginalized populations impacted by diabetes are at increased risk for poor health outcomes [16]; yet digital and technology-enabled interventions for diabetes self-management may help address the unique challenges faced by these communities and promote health equity. Three aspects of Pyx Health’s intervention may help explain its positive results, that is the process of matching patients to peer coaches with lived experience with uncontrolled diabetes [12], the focus on addressing SDoH that exacerbates diabetes poor health outcomes [17], and strong collaboration with a health plan. Specifically, telephone-based peer coaching has been shown to be effective at improving medication adherence among Black adults living in rural communities [18]. Thus, the matching process may have helped patients feel more comfortable endorsing a mix of behavioral and SDoH issues impacting their diabetes care including issues with appointments, disease self-management knowledge, and emotional health. Finally, MetroPlusHealth’s support, infrastructure, and endorsement helped engender trust in the peer program, which helped with member recruitment, ongoing engagement, and the overall positive impact of the program.

Evidence suggests that some low-income and minoritized patients do not feel comfortable enrolling and using digital diabetes self-management tools possibly due to low digital and tech literacy, which would impact their ability to download a mobile app, navigate a website, and create an account [19]. Further, these patients experience significant disparities in accessing digital health information [20], leading to potential distrust of digital-only solutions. Thus, establishing a telephone-based relationship with the peer coach may be preferable for Black and Latinx patients. In turn, developing trust with the peer coach can result in greater endorsement of SDoH concerns that would otherwise go unaddressed. This study provides evidence that digital health and peer-based programs for underserved populations can be successful when they account for digital literacy challenges and provide trusted support.

The value of addressing SDoH in health interventions cannot be overstated. There is a strong relationship between housing and food insecurity on worsening diabetes self-management and diabetes-related outcomes [10]; unfortunately, few digital health solutions adequately address SDoH despite the call to address social justice concerns [21]. The potential negative impact of not addressing SDoH is the perpetuation of health inequities and not realizing the full potential of digital tools. In this study, in addition to addressing knowledge of diabetes care and emotional health management, the most common issue addressed was managing medical appointments (including addressing barriers such as transportation, accessibility, and cost related to maintaining appointments). Further, nearly 50% (143/330) of patients endorsed housing and food insecurity as directly impacting their diabetes self-care. The results also suggest that endorsement of emotional health and medication adherence issues were related to a lack of improvement in HbA_1c_ relative to when these issues were not present in the patients’ lives. It is possible that comprehensive programs that address these SDoH barriers can result in health improvements.

This study provides important information regarding the role of technology-enabled peer coaching and addressing SDoH for patients with uncontrolled diabetes. Yet, the findings are not without limitations. First, the study does not include a randomly assigned control condition and thus causality could not be established. Future studies should consider a randomized controlled trial design in order to minimize the potential effect of confounding variables. Second, the COVID-19 pandemic impacted the process of program implementation and data collection, for example, patients were not able to continue routine HbA_1c_ testing due to social distancing mandates that may confound the outcomes of the program. This limitation also resulted in a smaller subsample of participants who had at least 2 HbA_1c_ laboratory test scores; thus, the analysis of factors attributed to changes in HbA_1c_ was exploratory and should be interpreted with caution. Nonetheless, it appears that patients who participated in the peer coaching program experienced greater reductions in HbA_1c_ and completed more HbA_1c_ monitoring than those not participating in the program. Further, digital coaching interventions for patients with type 2 diabetes have been found to be effective at improving health outcomes (eg, HbA_1c_, weight loss, fasting blood glucose, and BMI) ranging from 3 months up to 24 months; suggesting digital coaching programs have the potential to be effective and sustainable beyond 6 months [22]. In the long run, a reduction in clinical symptoms and improved disease monitoring may result in overall health improvements and a reduction of health care use costs. Finally, the sample demographics may not be generalizable to all settings, but we consider the overrepresentation of traditionally understudied groups as a strength, as well.

In conclusion, this analysis of a digital, remotely delivered 1-on-1 peer coaching program shows promise in improving diabetes self-management in a typically marginalized population with significant SDoH barriers. Program participants showed improved HbA_1c_ levels, and the analyses found that people with emotional stressors and medication management issues had worse outcomes and that many preferred to connect through phone calls versus an app. Altogether, these findings support the effectiveness of digital programs with multimodal approaches that include human support, showing success when they engage with empathy and address real-world issues including digital literacy and both BDoH and SDoH.

## Abbreviations

BDoH: behavioral determinants of health
CONSORT: Consolidated Standards of Reporting Trials
HbA1c: hemoglobin A1c
HIPAA: Health Insurance Portability and Accountability Act
SDoH: social determinants of health
